# Modelling, simulation, and experimental characterization of particle sedimentation inside a horizontal syringe

**DOI:** 10.1007/s10404-025-02802-x

**Published:** 2025-04-15

**Authors:** Maryamsadat Ghoreishi, Efsun Senturk, Gianluca Cidonio, Chiara Scognamiglio, Zita Salajková, Mara Riminucci, Alessandro Corsi, Giancarlo Ruocco, Marco Leonetti, Riccardo Reale

**Affiliations:** 1 Center for Life Nano- and Neuro- Sciences, Italian Institute of Technology, Rome, Italy; 2 Department of Mechanical and Aerospace Engineering, Sapienza University of Rome, via Eudossiana 18, 00184 Rome, Italy; 3 Institute of Nanotechnology of the National Research Council of Italy, CNR-NANOTEC, Rome Unit, Piazzale A. Moro 5, 00185 Rome, Italy; 4 Department of Civil Engineering and Computer Science, University of Rome Tor Vergata, Rome, Italy

**Keywords:** Bioengineering, Sedimentation, Syringe, 3D bioprinting, Lab-on-a-chip, Concentration half-life

## Abstract

Sedimentation is the settling of solid particles in a liquid medium driven by gravity. This phenomenon poses significant challenges in experimental lab-on-chip (LOC) applications, as they often involve a biological sample to be loaded inside a syringe for prolonged periods (e.g. 3D bioprinting, microfluidic cytometers). Mitigating solutions such as mechanical agitators or buffer adjustments exist, but increase the complexity and cost of the setup. In this work, we developed a model of particle sedimentation inside a horizontal syringe, which highlights the importance of several parameters: syringe radius, particle terminal velocity in the buffer, syringe outlet position, and flow-rate. The model provides a simple way to estimate the concentration half-life (${t}_{1/2}$), i.e. the time required for the concentration to halve, which is useful during the experiment design process. The model was initially tested numerically and then validated experimentally. Additionally, the applicability of the model to predict sedimentation of biological particles was experimentally demonstrated. Lastly, the model was used to develop guidelines for the design of setups with minimized sedimentation.

**Supplementary Information:**

The online version contains supplementary material available at 10.1007/s10404-025-02802-x.

## Introduction

Sedimentation, i.e. the process where particles in suspension settle out of the fluid in which they are originally contained due to an external force, has been encountered across a wide range of scientific fields throughout history, including biology, environmental engineering, chemistry, and pharmaceutical sciences (Grace et al. 1979). Recently, the gravity-driven sedimentation of particles inside a syringe has become a technologically relevant problem due to the widespread development of microfluidics and lab-on-chip (LOC) technologies (Puttrich et al. 2023). In fact, such technologies often require cells and particles to be suspended in a liquid buffer and loaded inside a syringe in order to be delivered into the system or chip by a syringe pump. The density mismatch between the loading buffer and the suspended particles results in a net gravitational force, leading to sedimentation. In these systems, once a particle reaches a syringe wall, it becomes stuck because fluid velocity in the proximity of walls is insufficient to further move it.

As an example, the sedimentation of cells in a bioink syringe reservoir poses significant challenges during 3D bio-printing. Due to the net gravitational force exerted on the cells, the suspended cells gradually settle to the bottom of the syringe, creating an uneven cell concentration (Xu et al. 2022; Mao et al. 2020). This phenomenon promotes the aggregation of cells, leading to an unstable printing process and potential nozzle clogging (Cho et al. 2011). This negatively affects the printing performance, resulting in significantly reduced functionality and viability of the printed tissues and organs (Xu et al. 2022; Graham et al. 2017; Bhattacharyya et al. 2021; Saunders et al. 2008). Similarly, sedimentation is known to negatively impact the performance of microfluidic cytometers and cell sorters. When left in a syringe for an extended period of time, cells will sediment, resulting in wrong estimates of sample concentration and lowering system throughput. Additionally, as larger cells tend to sediment faster than smaller ones (cfr. Equation 1), they will sediment preferentially, resulting also in wrong estimates of relative concentrations (e.g. leukocytes differential count) (Freyer et al. 1989).

Two approaches to minimize particle sedimentation in syringes for LOC systems have been proposed: mechanical agitators, and buffer adjustments. Mechanical agitation systems aim to introduce turbulence in the syringe with stirring or shaking devices. This class features both commercial instruments (Cetoni 2024; Gpd-global 2024) as well as research prototypes (Puttrich et al. 2023), but results in a setup which is more complex, more expensive, and less portable. Sedimentation can also be mitigated by modifying the suspension buffer (Barabè et al. 2020). By adding a solute, the buffer-cells density mismatch which drives the sedimentation can be drastically reduced. As the buffer (usually phosphate-buffered saline) must remain isotonic to avoid cell rupture or shrinkage, larger molecules (e.g. sucrose) are usually adopted (Martin et al. 2006; Reale et al. 2019). Changing the suspension viscosity is another known option to decrease sedimentation. For example, the addition of Xanthan gum increases buffer viscosity and mitigates sedimentation, even though it leads to higher shear stresses experienced by the cells, potentially impacting their viability (Freyer et al. 1989). Thus, while solutions to mitigate sedimentation exist, they increase the complexity or reliability of the system.

In this work, we aim to quantify the sedimentation dynamic of particles inside a horizontal syringe, and to provide an easy way to estimate the time required for the initial concentration to halve, i.e. the concentration half-life ${t}_{1/2}$. This parameter quantifies the sedimentation to be expected during the design of a new experiment involving a sample loaded into a horizontal syringe and informs on whether a mitigation approach will be required. Firstly, a sedimentation model is developed, highlighting the parameters controlling sedimentation (namely, particle terminal velocity, syringe radius, and flow-rate) and providing estimates for ${t}_{1/2}$. Successively, the sedimentation model is tested using numerical Finite Element Modelling (FEM) simulations and validated experimentally using polymeric particles. We further demonstrate the applicability of the model to biological targets by predicting the sedimentation of primary human bone marrow stromal cells (HBMSCs), pivotal for the engineering of skeletal tissue substitutes for tissue engineering. Lastly, we use the model to provide guidelines aimed at minimizing sedimentation.

### Sedimentation inside a horizontal syringe

**Fig. 1:**
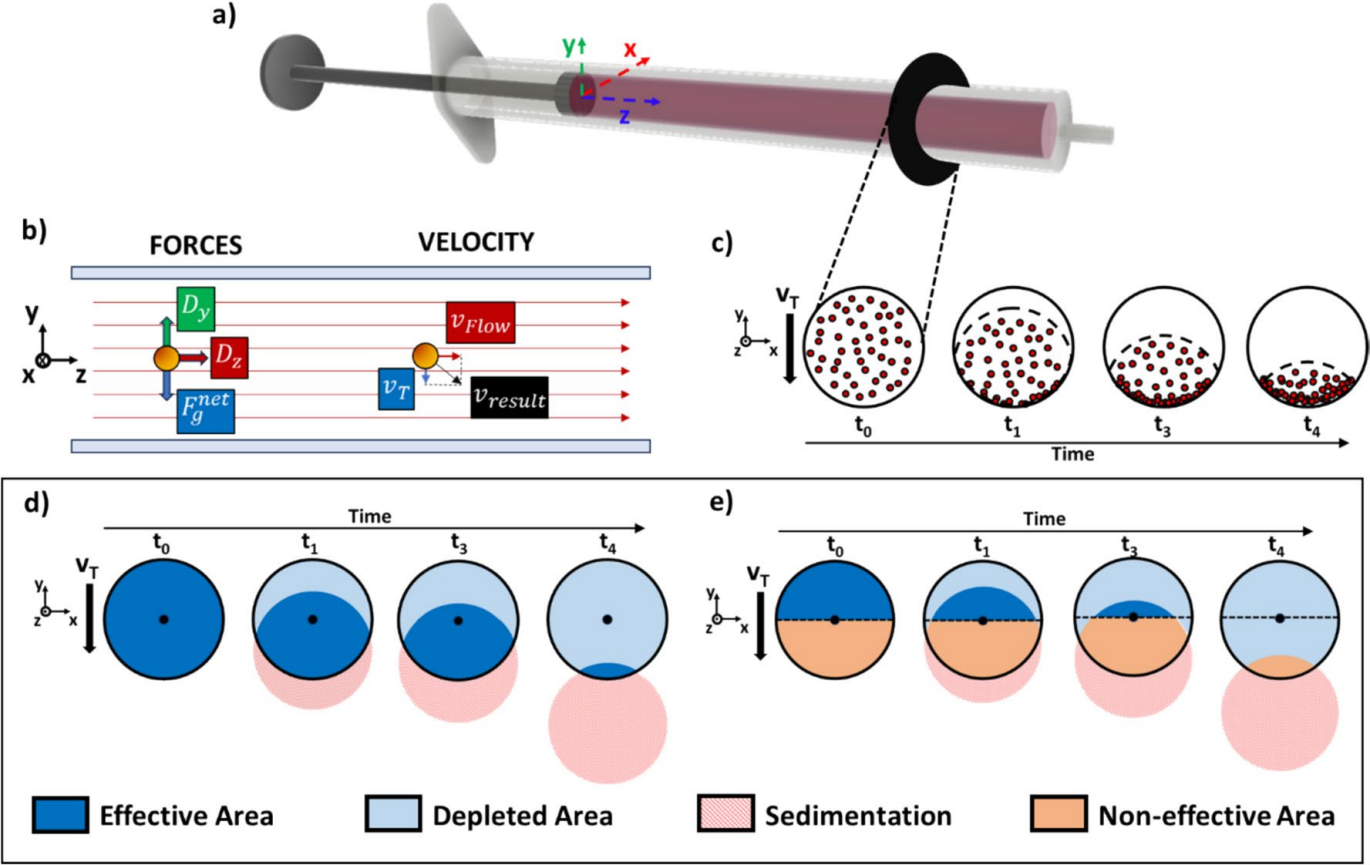
Sedimentation inside a horizontal syringe. **a**, **b** System model: the forces acting on particles are on particles are gravity, ${F}_{g}^{net},$ and the fluid drag (both horizontally along the z-axis, ${D}_{Z},$ and vertically along the y-axis, ${D}_{y}$). **c** In the syringe cross-section, sedimentation causes a downward translation of particles over time. If all particles move at the same velocity, they maintain their relative positions to each other, and the local concentration remains constant. **d** As particle undergo sedimentation, they leave behind a region depleted of particles which results in a decreased effective concentration. **e** If the vertical component of the buffer velocity field is lower than particle terminal velocity everywhere in the syringe, particles below the nozzle will never be able to leave the syringe, creating a non-effective region, which is a region filled with particles that do not count towards the effective concentration

Sedimentation derives from a density difference between the particle and the buffer, resulting in a net gravitational force acting on the particle (Fig. 1). Along the vertical axis, particles accelerate until the gravitational force is counterbalanced by the drag force of the surrounding fluid. At the point of equilibrium, particles reach a stable terminal velocity ${v}_{T}$ (Nouri et al. 2014), which for an ideal sphere in a laminar flow can be determined by imposing the net gravitational force to be equal to the Stokes’ drag (Stokes 2009; Lamb 1994):

1 $$\begin{array}{c}{v}_{T}=\frac{2g{r}_{P}^{2}\left({\rho }_{B}-{\rho }_{P}\right)}{9\eta }\end{array}$$

where $g$ is the gravitational acceleration, ${\rho }_{B}$ is the density of the buffer, ${\rho }_{P}$ is the density of the particle, $\eta$ is the fluid viscosity and ${r}_{P}$ is the particle radius. If particles are lighter than the buffer (${\rho }_{B}>{\rho }_{P}$) they will float to the top (${v}_{T}> 0$), whereas if they are heavier (${\rho }_{B}<{\rho }_{P}$) they will sediment towards the bottom (${v}_{T}<0$).

While several sedimentation models have been previously presented in the literature, none of them describes the case of sedimentation inside a syringe with its distinguishing features: the presence of an underlying flow-field, a cylindrical geometry, and a small outlet. For example, simple sedimentation models (Cremaschini et al. 2024; Lane et al. 2019) recognize that the Stokes’ drag is the driving force of sedimentation, but don't provide estimates for the concentration half-life, whereas models in a microchannel (Pinho et al 2017) fail to account for the cylindrical syringe geometry. Additional models investigating more sophisticated aspects of sedimentation have also been proposed: Liu’s group (Liu et al. 2015) described a mesoscale approach to study of particle sedimentation with inertia effect at higher Reynolds’ numbers between two parallel walls and Cölfen’s group (Cölfen and Lucas 2006) investigated the effects of buffer pH on particle sedimentation.

In LOC applications, particles can be assumed to move at a constant downward velocity, as they are heavier than their surrounding buffer and their acceleration time required to reach ${v}_{T}$ is in the order of milliseconds (Fig. S1). Particles move with velocity ${v}_{T}$ until they reach the bottom of the syringe, where they stick to the wall and are no longer in suspension (Fig. 1c). As a result, the number of particles still in suspension in the syringe, i.e. the sample effective concentration ${c}_{E}\left(t\right)$, decreases over time. To quantify this process, it is useful to analyse the syringe cross-section, where the region occupied by particles, i.e. the effective region ${A}_{E}\left(t\right)$, decreases over time, leaving behind a particle-depleted region (Fig. 1d). If all particles move with the same downward velocity ${v}_{T}$, their relative positions to each other are constant and all particles behave like a unique system undergoing a vertical translation. Thus, no accumulation takes place inside the syringe bulk and the local concentration in the effective region remains constant and equal to the initial concentration ${c}_{0}$. The decrease in effective concentration ${c}_{E}\left(t\right)$ is due to the decrease in the volume of the effective region, ${V}_{E}\left(t\right)$:

2 $$\begin{array}{c}{c}_{E}\left(t\right)=\frac{N\left(t\right)}{{V}_{s}}= {c}_{0}\frac{{V}_{E}\left(t\right)}{{V}_{s}}={c}_{0}\frac{{A}_{E}\left(t\right) {L}_{s}}{{A}_{S} {L}_{s}}={c}_{0}\frac{{A}_{E}\left(t\right)}{{A}_{S}}\end{array}$$

where $N\left(t\right)$ is the number of particles in suspension over time, and ${V}_{s}$, ${A}_{S}$, and ${L}_{S}$ are the syringe volume, cross-sectional area, and length, respectively. It follows:

3 $$\begin{array}{c}\frac{{c}_{E}\left(t\right)}{{c}_{0}}=\frac{{A}_{E}\left(t\right)}{{A}_{S}}\end{array}$$

The sedimentation dynamics is described by the evolution of the area of the effective region, ${A}_{E}$ (Fig. 1d), which can be computed as the intersection of two circles with the same radius, ${R}_{S}$, with a distance ${d}_{T}$ between their centers (Fig. S2) (Weisstein 2024):

4 $$\begin{array}{c}{A}_{E}=2 {R}_{S}^{2}{\text{cos}}^{-1}\left(\frac{{d}_{T}}{2 {R}_{S}}\right)-\frac{1}{2} {d}_{T} \sqrt{4 {R}_{S}^{2}-{d}_{T}^{2}}\end{array}$$

where the distance ${d}_{T}$ is the path travelled over time by particles due to sedimentation:

5 $$\begin{array}{c}{d}_{T}={v}_{T} t\end{array}$$

Substituting, Eq. 4 results in:

6 $$\begin{array}{c}{A}_{E}(t)=2 {R}_{S}^{2}{\text{cos}}^{-1}\left(\frac{{v}_{T} t}{2 {R}_{S}}\right)-\frac{1}{2} {v}_{T} t \sqrt{4 {R}_{S}^{2}-{\left({v}_{T} t\right)}^{2}}\end{array}$$

Further substituting Eq. 6 in Eq. 3 (considering that ${A}_{S}=\pi {R}_{S}^{2}$), it is obtained:

7 $$\begin{array}{c}\frac{{c}_{E}\left(t\right)}{{c}_{0}}=\frac{{A}_{E}\left(t\right)}{{A}_{S}}=\frac{2 {R}_{S}^{2}{\text{cos}}^{-1}\left(\frac{{v}_{T} t}{2 {R}_{S}}\right)-\frac{1}{2} {v}_{T} t \sqrt{4 {R}_{S}^{2}-{\left({v}_{T} t\right)}^{2}}}{\pi {R}_{S}^{2}}\end{array}$$

which simplifies into:

8 $$\begin{array}{c}\frac{{c}_{E}\left(t\right)}{{c}_{0}}=\frac{2 }{\pi }{\text{cos}}^{-1}\left(\frac{{v}_{T} t}{2 {R}_{S}}\right)- \frac{1}{2\pi }\frac{ {v}_{T} t }{{R}_{S}^{2}}\sqrt{4 {R}_{S}^{2}-{\left({v}_{T} t\right)}^{2}}\end{array}$$

which is dimensionless and quantifies the sedimentation process over time. It returns, as expected:

$$t= 0 ; \frac{{C}_{E}}{{C}_{0}}=1$$

$$t= \frac{2 {R}_{S}}{{v}_{T}}; \frac{{C}_{E}}{{C}_{0}}=0$$

indicating that the sedimentation is complete when a sufficient time is elapsed for particles to travel a path equal to the syringe diameter. From an experimental point-of-view, it can be useful to consider the time required for the concentration to halve, i.e. the concentration half-life ${t}_{1/2}$ to design the experiment, which can be computed numerically from Eq. 8, resulting in:

9 $$\begin{array}{c}{t}_{1/2}\sim 0.8\frac{ {R}_{S}}{{v}_{T}}; \frac{{C}_{E}}{{C}_{0}}=\frac{1}{2}\end{array}$$

Equation 4–9 consider all particles in suspension to be effective. Nevertheless, in most syringes the nozzle is located in the centre of the cross-section. Consequently, in the absence of other vertical velocity components, particles in the lower half of the syringe will never be able to reach the syringe nozzle and exit the syringe. As shown in Fig. 1e, this creates a non-effective region, which is occupied by particles that are in suspension but that will not be able to reach the outlet and leave the syringe. In this case, the area of the effective region, ${A}_{E}$, can be computed as the area of a circular segment:

10 $$\begin{array}{c}{A}_{E}={R}_{S}^{2}{\text{cos}}^{-1}\left(\frac{{d}_{T}}{{R}_{S}}\right)-{d}_{T} \sqrt{{R}_{S}^{2}-{{d}_{T}}^{2}}\end{array}$$

Using again Eqs. 3 and 5, and considering that in this case the initial effective area is ${A}_{S} = \pi {R}_{S}^{2}/2$ (i.e. only the region above the nozzle is effective), Eq. 8 becomes:

11 $$\begin{array}{c}\frac{{c}_{E}\left(t\right)}{{c}_{0}}=\frac{{A}_{E}\left(t\right)}{{A}_{S}}=\frac{{R}_{S}^{2}{\text{cos}}^{-1}\left(\frac{{v}_{T} t}{{R}_{S}}\right)-{v}_{T} t \sqrt{{R}_{S}^{2}-{\left({v}_{T} t\right)}^{2}}}{\frac{\pi {R}_{S}^{2}}{2}}\end{array}$$

which simplifies into:

12 $$\begin{array}{c}\frac{{c}_{E}\left(t\right)}{{c}_{0}}={\frac{2}{\pi }\text{cos}}^{-1}\left(\frac{{v}_{T} t}{{R}_{S}}\right)-\frac{2}{\pi } \frac{{v}_{T} t}{{R}_{S}^{2}} \sqrt{{R}_{S}^{2}-{\left({v}_{T} t\right)}^{2}}\end{array}$$

which is dimensionless and returns:

$$t= 0 ;\,\, \frac{{C}_{E}}{{C}_{0}}=1$$

$$t= \frac{{R}_{S}}{{v}_{T}};\,\, \frac{{C}_{E}}{{C}_{0}}=0$$

indicating that in this case the sedimentation is complete when a sufficient time is elapsed for particles to travel a path equal to the syringe radius. Similarly to Eq. 9 the concentration half-life ${t}_{1/2}$ can be computed numerically to be:

13 $$\begin{array}{c}{t}_{1/2}\sim 0.4 \frac{{R}_{S}}{{v}_{T}}; \frac{{C}_{E}}{{C}_{0}}=\frac{1}{2}\end{array}$$

Equations 8 and 12, are derived under the assumption that no other vertical forces act on particles, and highlight that the sedimentation dynamic depends only on particle terminal velocity and on the syringe radius. The assumption holds true for most of the syringe length but not close to nozzle, where fluid streamlines converge towards the outlet (Watson et al.^1986^; Syntouka et al. 2018). In this region, the fluid also has a vertical velocity component which can affect the sedimentation process. If the resulting vertical particle velocity is positive (${v}_{Flow}^{y}+{v}_{T}>0$), particles will be lifted towards the nozzle and leave the syringe; otherwise (${v}_{Flow}^{y}+{v}_{T}<0$) they will sediment to the bottom of the syringe. Thus, the underlying flow field must be fully characterized to accurately compute particle trajectories, usually requiring tailored FEM simulations as the computation depends on the syringe geometry and the applied flow-rate (Fig. S3).

**Fig. 2:**
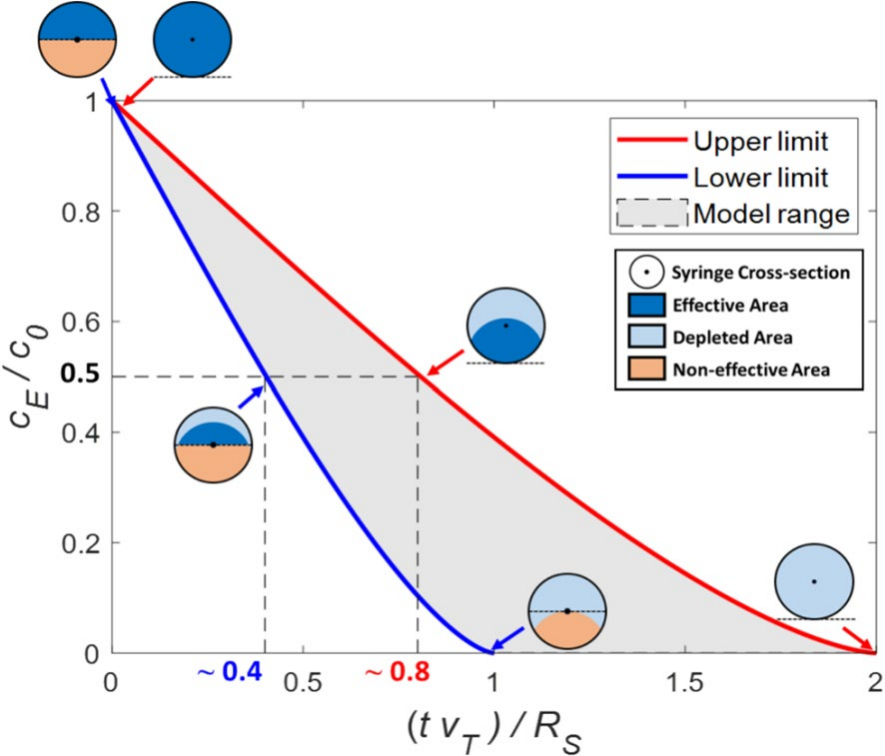
Sedimentation dynamics according to the proposed model. The model describes the decrease in effective concentration ${C}_{E}/{C}_{0}$ as a function of the time $t$, the terminal velocity ${v}_{T}$, and the syringe radius ${R}_{S}$. The red line represents the upper boundary described by Eq. 8, whereas the the blue line represents the lower boundary described by Eq. 12. The intersection between the line ${C}_{E}/{C}_{0}=0.5$ and the two boundaries yield the values for ${K}_{S}$ given in Eq. 15. The shaded gray area between the boundaries represents the range allowed by the model. By increasing the flow-rate, the sedimentation dynamics moves from the blue line to the red line. Syringe cross-section schematics show the effective/depleted/non-effective areas for ${C}_{E}/{C}_{0}=1$, 0.5, and 0 for the two conditions

Nevertheless, from an experimental point-of-view, the accurate quantification of the sedimentation process is not always necessary, and an estimate of its timescale is sufficient to design experiments. In particular, Eqs. 9 and 13 represent the two limiting-cases for the concentration half-life (Fig. 2):

14 $$\begin{array}{c}\frac{0.4 {R}_{S}}{{v}_{T}}<{t}_{1/2}<\frac{0.8 {R}_{S}}{{v}_{T}}\end{array}$$

Or:

15 $$\begin{array}{c}{t}_{1/2}={K}_{S}\frac{{R}_{S}}{{v}_{T}};\,\, 0.4<{K}_{S}<0.8\end{array}$$

where ${K}_{S}$ is an empirical sedimentation constant which depends on the system flow-rate. The lower boundary represents the condition where ${v}_{Flow}^{y}+{v}_{T}<0$ everywhere in the syringe, usually observed for low flow-rates: in this condition, all particles below the nozzle are lost, as nowhere is the fluid able to lift them. The upper boundary represents the condition where ${v}_{Flow}^{y}+{v}_{T}>0$ in the cross-section near the nozzles, usually observed for high flow-rates: in this condition, the fluid will lift all particles in the section of the syringe near the nozzle, irrespectively of their cross-sectional position. Therefore, even though the flow-rate does not explicitly appear in Eq. 14, it affects sedimentation by controlling the system behaviour between the two boundaries.

## Materials and methods

### Numerical analysis

**Fig. 3:**
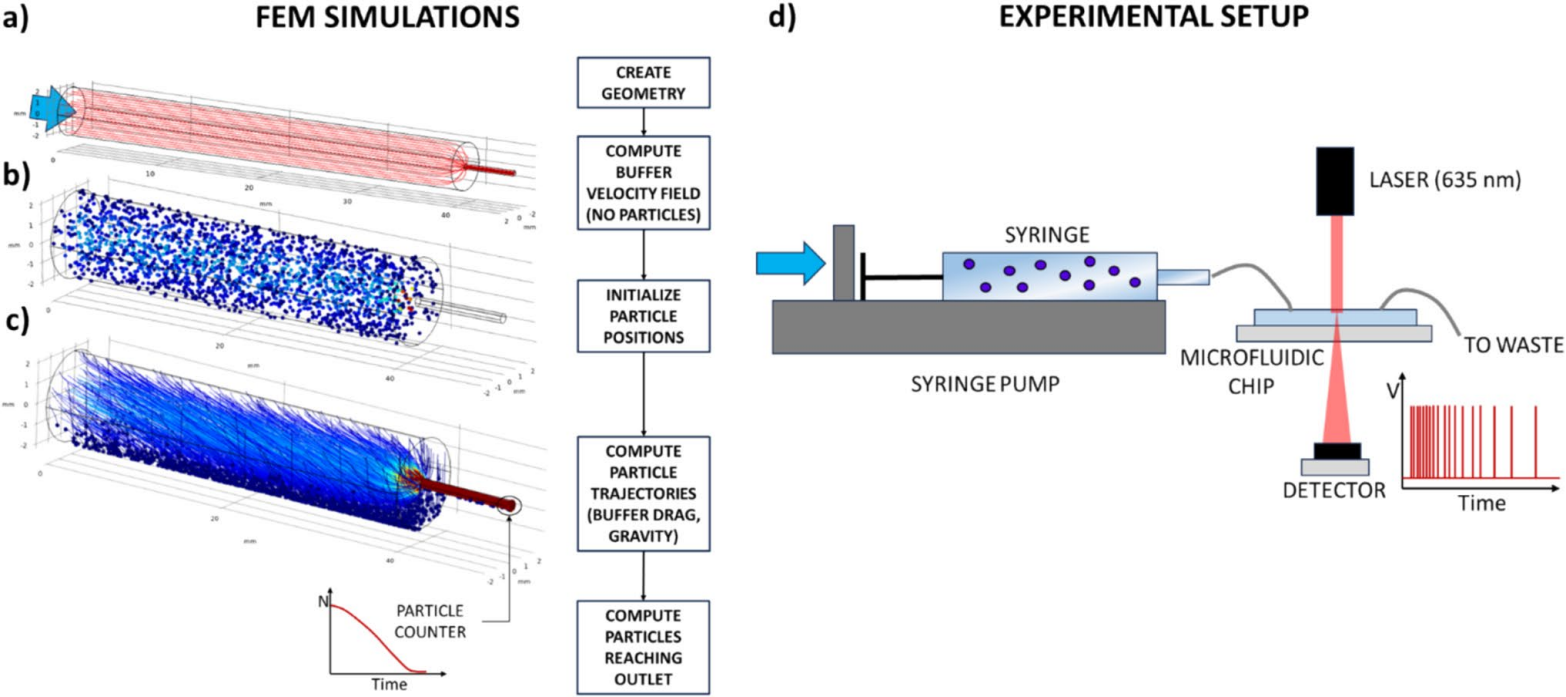
Sedimentation model validation. **a**–**c** FEM simulations were performed using COMSOL Multiphysics®. **a** After creating the syringe FEM model, the velocity field of the buffer without particles is computed. **b** 2000 particles are randomly generated inside the syringe. **c** The velocity field is used to calculate particle trajectories while subject to gravity and fluid drag. Lines represent particle trajectories with their color dependindg on the local velocity. The number of particles reaching the outlet over time is measured with a particle counter and used to quantify sedimentation. **d** Experimental setup: samples are loaded onto a syringe mounted on a syringe pump and connected to a microfluidic particle counting chip. A laser is focused on the measurement region of the microfluidic chip: when a particle passes through the measurement region it interacts with the laser, causing a peak in the signal measured by the detector. The frequency of particles passing through the counter is used to quantify sedimentation

To validate the sedimentation model, a finite element model (FEM) of particle sedimentation inside a horizontal syringe was created using COMSOL Multiphysics®, according to the workflow in Fig. 3-a: (i) The 3D syringe geometry was recreated: a main cylinder (${R}_{S}$= 2.2 mm, L = 40 mm) represented the syringe body and a secondary cylinder (${R}_{O}$= 0.5 mm, L = 5 mm) located at the centre of the main one represented the syringe outlet. The domain was discretized with a free tetrahedral mesh with 20,119 elements (average element quality = 0.68, minimum element quality = 0.218); (ii) The buffer steady-state velocity field was computed using the creeping flow module to calculate the fluid streamlines inside the syringe in the absence of particles. Water at room temperature was used as the buffer, and additional simulations were performed for different buffer viscosities (η = 0.7, 1, 1.3, 1.6, and 2 mPa s). Boundary conditions used include: no-slip on the side walls, atmospheric pressure at the outlet, and constant flow-rate (Q = 0.5, 1, 5, 10, 15, 20, 30, and 50 μl/min) on the back of the syringe, representing the syringe plunger being pushed by the pump actuator. (iii) For each condition, 2,000 particles were initialized inside the syringe with randomized positions. Simulations were performed for different particle size (${r}_{P}=$ 2, 3, 4, 5, 6, and 7.5 μm) and densities (${\rho }_{P}$= 1.01, 1.015, 1.035, 1.07, 1.10, 1.13, 1.19 g/cm^3^), and for syringes with different radii (${R}_{S}$= 1.15, 1.625, 2.3, and 3.6 mm) and outlet position (concentric, eccentric top, eccentric bottom). (iv) Particle trajectories were computed using the built-in particle tracking module (0.05 s time-step). Particles were subject to gravity and drag. The buffer flow previously calculated was used as velocity field. Effects of particles on the fluid flow were considered negligible, as particles involved in LOC studies are usually small (< 50 μm) and diluted (< 10^7 ^particles/ml). (v) The cumulative number of particles leaving the syringe over time was monitored by a particle counter at the syringe outlet and used to compute the effective concentration over time.

### Experimental setup

The sedimentation model was validated experimentally measuring the sedimentation of polymeric particles and mammalian cells with a microfluidic particle counter. As shown in Fig. 3-d, samples were loaded onto a syringe mounted on a syringe pump (Elite 2000, Harvard Apparatus) and connected to a counting chip (a modified version of a previously reported microfluidic scanning flow cytometer) (Reale et al. 2023). A laser (635 nm, 5 mW, RS Components) was focused on the measurement region of the microfluidic chip: when a particle passes through the measurement region interacts with the laser, scattering light which is collected by a photoreceiver (APD410A2/M – Si, Thorlabs) and causing a peak in the recorded signal. The microfluidic chip is made of polydimethylsiloxane (PDMS) using standard soft-lithography, with a measurement region formed by a straight channel (width = 120 μm, height = 25 μm) and sheath flow channels to focus particles at the centre of the measurement region (Fig. S4). Signals were acquired with a USB data acquisition card (USB-6009, National Instruments). The number of particles counted over 1 s intervals was used to estimate the effective concentration. Three independent replicates were performed for each experimental condition.

### Sample preparation

Polymeric beads with different size (${r}_{P}=$ 3, 5, and 7.5 μm) and materials were purchased from Spherotech, Inc. (Polystyrene, PS) and Microparticles, GmbH (Poly(methylmethacrylate, PMMA). Each bead type was dissolved in Phosphate Buffer Saline (PBS, η = 1 mPa s) with a concentration of approximately ${10}^{6}$ particles/ml, and PBS was used as the sheath flow. Samples were loaded into the 1 ml glass syringe (Hamilton, d = 4.6 mm) for the main flow, while a plastic 10 ml syringe (Fisherbrand) was used for the sheath flow. To analyse the effects of flow-rate on sedimentation, the main sample was pumped at 10, 15, and 20 μl/min while maintaining a fixed flow-rate of 30 μl/min for the sheath flow. Human Bone Marrow Mesenchymal Stem Cells (HBMSCs) were isolated from healthy donors as previously described (Robey et al. 2014) and used in this study according to the Declaration of Helsinki and approved (June 22nd, 2023) by the Institutional Review Board (Department of Molecular Medicine, Sapienza University of Rome, Italy). HBMSCs were cultured in Alpha Minimum Essential Medium (Merck-Sigma) prepared with 1% Penicillin/ Streptomycin (v/v), 10% Fetal Bovine Serum (v/v) at 37 °C, 5% CO_2_. 5 M/ml cells were collected with using trypsin–EDTA (Merck-Sigma, Italy) when they were 80% confluent for quantification experiment. Cells were dissolved in the PBS with a concentration of approximately ${10}^{6}$ particles/ml, and PBS was used as the sheath flow. The contribution of suspended particles/cells to the final viscosity of both samples was computed to be negligible (< 10^–3^ mPa s) using Einstein's relation (Mardles 1940).

### Data analysis

Experimental and simulation data were analysed with a custom Matlab script. The experimental signal deriving from the photodetector was filtered with a low-pass filter with 100 Hz cut-off frequency to remove high frequency noise, and a peak-finding procedure was used to measure the number of particles that passed through the measurement region every second. The throughput curve was normalized with respect to the initial value to obtain the ratio ${C}_{E}/{C}_{0}$, filtered with a low-pass filter, and ${t}_{1/2}$ measured (Fig. S5).

## Results and discussion

The proposed sedimentation model was tested with FEM simulations and validated experimentally. The concentration half-life ${t}_{1/2}$ was simulated and measured under different conditions and compared with estimates obtained using Eq. 15. Firstly, using polymeric particles, the dependency of sedimentation on terminal velocity. Successively, the applicability of the model to biological samples was verified by comparing estimate ${t}_{1/2}$ for mammalian cells with experimental values. Lastly, the effects of the flow-rate on sedimentation were analysed.

### Effects of terminal velocity on concentration half-life

Experiments and simulations with different particle terminal velocities were performed by independently changing each parameter in Eq. 1 (i.e. particle size, particle-buffer density difference, and buffer viscosity), while using a constant flow-rate (Q = 10 μl/min) and syringe radius (${R}_{S}$= 2.3 mm). For each condition, the sedimentation curve was measured/simulated and the concentration half-life ${t}_{1/2}$ computed. The effects of particle size were assessed by quantifying the sedimentation of PS beads with different radii, namely ${r}_{P}$= 2, 3, 4, 5, 6, and 7.5 μm for simulations, and ${r}_{P}$= 3, 5, and 7.5 μm for experiments. The effects of particle density were assessed by quantifying the sedimentation of beads with the same size (${r}_{P}$= 5 μm) and different densities, namely ${\rho }_{P}$=1.01, 1.015, 1.035, 1.07, 1.10, 1.13, 1.19 g/cm^3^ for simulations, and ${\rho }_{P}$=1.07 g/cm^3^ (PS) and ${\rho }_{P}$=1.19 g/cm^3^ (PMMA) for experiments. The effects of buffer viscosity were assessed by simulating the sedimentation of PS beads (${r}_{P}$= 5 μm) in a buffer with different dynamic viscosities, namely η = 0.7, 1, 1.3, 1.6, and 2 mPa s.

**Fig. 4:**
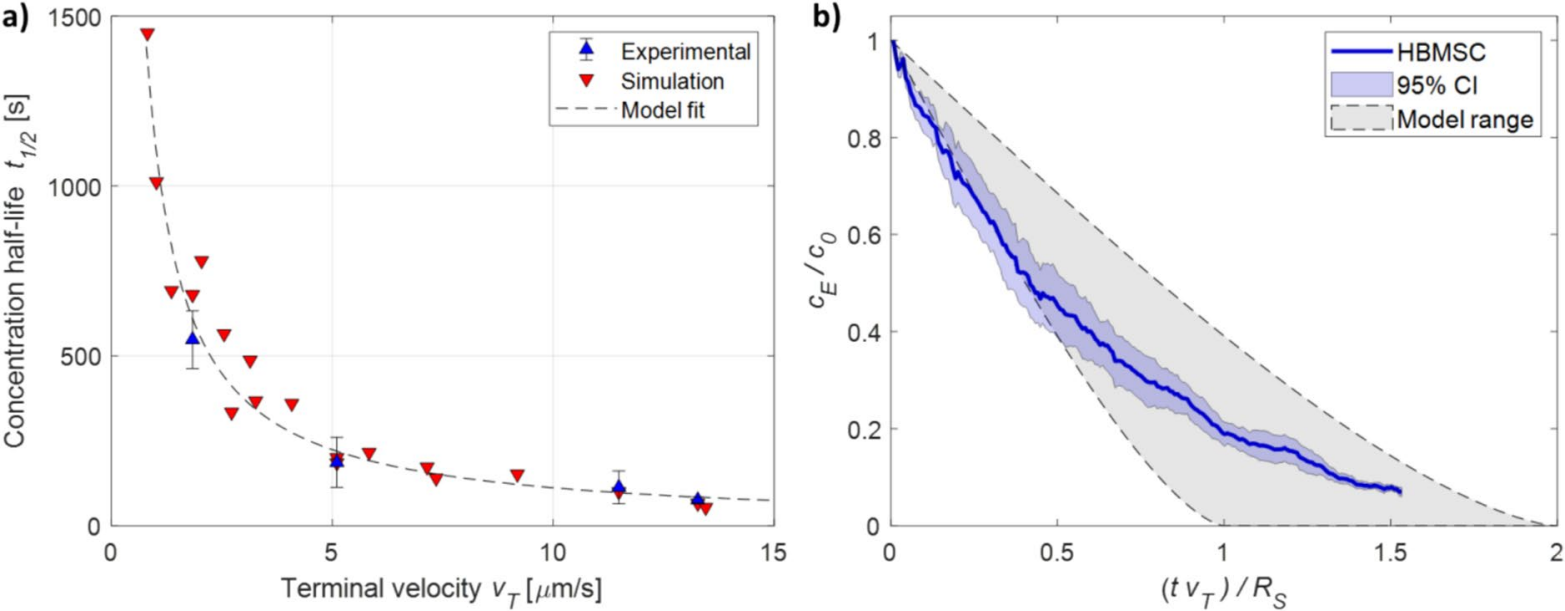
Sedimentation model validation. **a** Concentration half-life ${t}_{1/2}$ versus terminal velocity ${v}_{T}$*.* Red downward triangles represent FEM simulations, blue upward triangles represent experimental data on polymeric particles. Error bars represent standard deviation (*n* = 3). The dashed line represents the best fit using Eq. 15, yielding ${K}_{S}=0.52$ and *R*^2^ = 0.94. **b** Applicability of the sedimentation model to a biological target. The solid blue line represents the experimental sedimentation of mammalian cells (HBSMC), with the blue shaded area representing 95% confidence intervals (*n* = 3). The gray shaded area represents the range predicted by Eq. 15

The effects of each parameter were quantified independently by fitting simulated/measured values with the appropriate function obtained by substituting Eq. 1 in Eq. 15 (Fig. S6), yielding R^2^ = 0.99 for ${t}_{1/2}\propto {r}_{P}^{-2}$, R^2^ = 0.95 for ${t}_{1/2}\propto {\left({\rho }_{B}-{\rho }_{P}\right)}^{-1}$, and R^2^ = 0.99 for ${t}_{1/2}\propto \eta$. The terminal velocity ${v}_{T}$ was computed for each simulation/experiment previously described and all data were pooled and fitted to ${t}_{1/2}$ according to Eq. 15. As shown in Fig. 4a, the model successfully fit the data with an R^2^ = 0.94. The fit returned a constant of ${K}_{S}=0.5$ 2, which is within the boundaries predicted by the proposed model (Eq. 15).

### Prediction of concentration half-life for biological targets

To verify if the proposed model can also predict the sedimentation of biological targets, the sedimentation of HBMSCs was measured experimentally (Fig. 4b). Measurements yielded ${t}_{1/2}=61\pm 18$ s (n = 3), in good agreement with the model estimate of ${t}_{1/2}$=63 s, obtained using size and density values from the literature (${r}_{P}$= 10.3 μm, ${\rho }_{P}$=1.062 g/cm^3^) (Drobek et al. 2023).

### Effects of flow-rate on sedimentation constant and concentration half-life

**Fig. 5:**
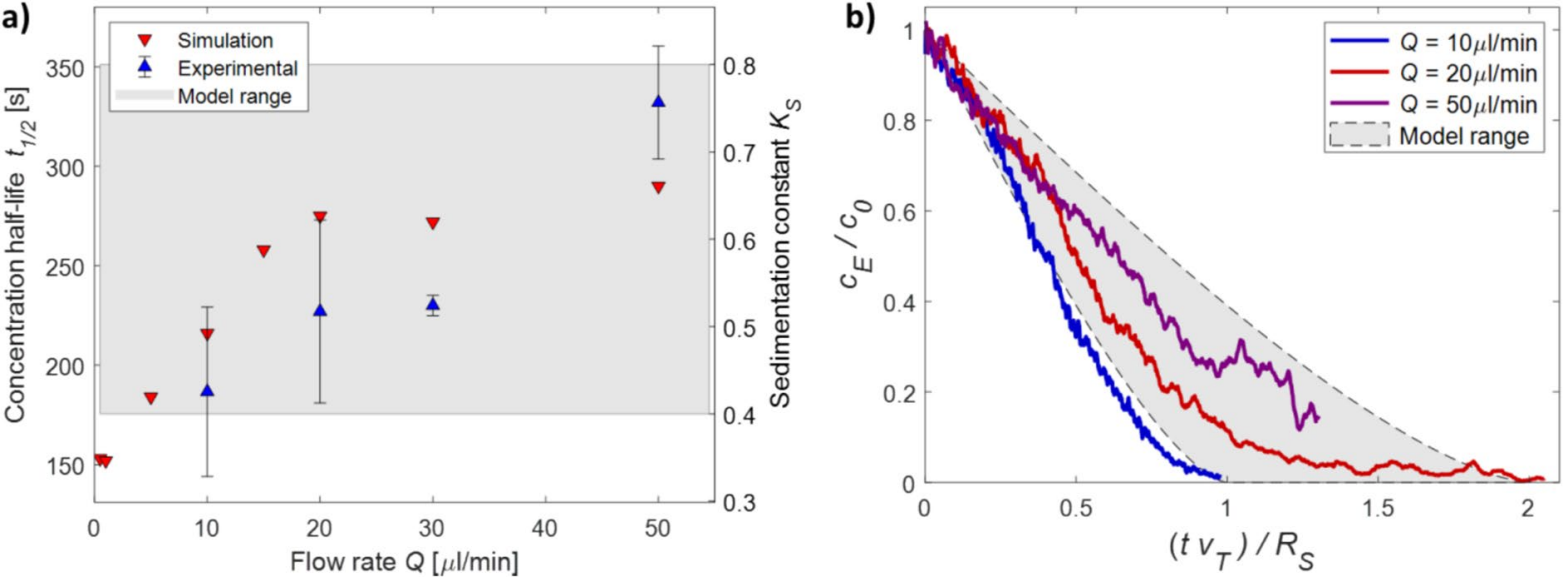
Effects of flow-rate on concentration half-life ${t}_{1/2}$. In both plots, the shaded gray area represents mode predicted range according to Eq. 15. **a** Simulation and experimental data at increasing flow-rate. Red downward triangles represent FEM simulations, and blue upward triangles represent experimental data on polymeric particles. Errorbars represent standard error of the mean (*n* = 3). The model is used to compute the sedimentation constant, which is reported on the right y-axis. **b** Experimental sedimentation of polymeric particles at increasing flow-rate (blue line *Q* = 10 μl/min, red line *Q* = 20 μl/min, purple line *Q* = 50 μl/min)

According to the proposed model, the flow-rate affects the velocity field inside the syringe and determines the areas where ${v}_{Flow}^{y}+{v}_{T}>0$, thus affecting the multiplicative constant ${K}_{S}$ and consequently ${t}_{1/2}$. The effects of flow-rate on the sedimentation were assessed by quantifying the sedimentation of PS beads (${\rho }_{P}$=1.07 g/cm^3^, ${r}_{P}$= 5 μm) under different flow-rates, namely $Q$ =0.5, 1, 5, 10, 15, 20, 30, 50 μl/min for simulations and $Q$ =10, 20, 30, and 50 μl/min for experiments. For each condition, the concentration half-life ${t}_{1/2}$ was obtained from the measured/simulated sedimentation curve. As shown in Fig. 5-a, an increase in the flow-rate resulted in an increase in concentration half-life in simulations, from ${t}_{1/2}=153 s$ at $Q$ =0.5 μl/min to ${t}_{1/2}=$ 290 s at $Q$ =50 μl/min. The trend was confirmed experimentally, with ${t}_{1/2}$ increasing from ${t}_{1/2}=187\pm 42$ s at $Q$=10 μl/min to ${t}_{1/2}=$ 332 + 28 s at $Q$=50 μl/min. The corresponding values of the sedimentation constant ${K}_{S}$ obtained using Eq. 15, are in the range $0.35<{K}_{S}<0.66$ for simulations and $(0.42\pm 0.09)<{K}_{S}<(0.76\pm 0.06)$ for the experiments, in good agreement with the model range of $0.4<{K}_{S}<0.8$. As shown in Fig. 5-b, experimental data confirmed the model prediction that the flow-rate affects the sedimentation behaviour of the system, moving the sedimentation curve from the lower towards the upper boundary. Thus, using a high flow-rate it is possible to significantly increase the concentration half-life, yielding up to a ~ twofold increase with respect to low flow-rate conditions.

### Effects of syringe geometry on concentration half-life

**Fig. 6:**
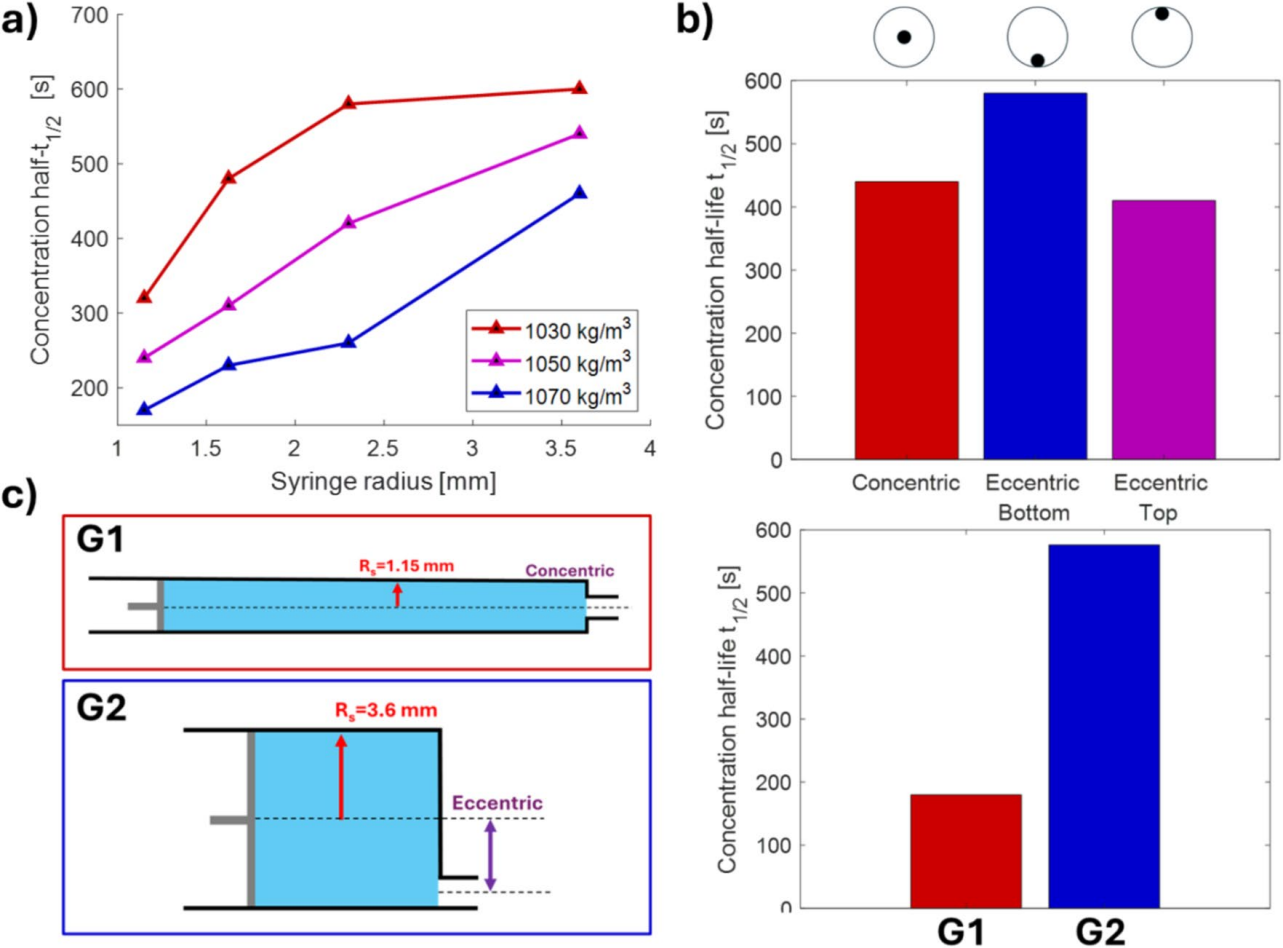
Simulated effects of syringe geometry on concentration half-life ${t}_{1/2}$. **a** Effects of syringe radius on sedimentation dynamics of particles (*ρ*_*P*_ = 1.07, 1.05, and 1.03 g/cm3, *r*_*P*_ = 5 µm). **b** Effects of syringe outlet position on sedimentation dynamics of particles (${\rho }_{P}$=1.07 g/cm3, ${r}_{P}$= 5 µm). **c** Comparison of sedimentation dynamics of particles (${r}_{P}$= 5 µm, ${\rho }_{P}$=1.07 g/cm3) in syringes with standard geometry (G1, a concentric syringe with ${R}_{S}$=1.15 mm corresponding to a 250 µl syringe) and an optimized syringe (G2, an eccentric syringe with ${R}_{S}$=3.6 mm corresponding to a 2.5 ml syringe)

Simulations were performed to investigate how syringe geometrical parameters (namely the syringe barrel radius ${R}_{S}$ and the outlet position) affect the concentration half-life. The syringe barrel radius ${R}_{S}$ appears explicitly in Eq. 15, which predicts that larger syringe radii should lead to increased ${t}_{1/2}$. This was confirmed by simulating the sedimentation dynamics of particles of different densities (${\rho }_{P}$=1.07, 1.05, and 1.03 g/cm^3^, ${r}_{P}$= 5 μm) in syringes with different radii, namely ${R}_{S}$ =1.15, 1.625, 2.3, and 3.6 mm (corresponding to typical syringes with volume 0.250, 0.5, 1, and 2.5 ml, respectively). The concentration half-life increased with the syringe radius for each simulated condition, as shown in Fig. 6-a. It is worth noting that while a greater syringe radius leads to a reduction in cross-sectional velocity (i.e. the constant ${K}_{S}$ in Eq. 15), the impact of this detrimental effect is smaller than the benefits yielded by the longer radius, resulting in a net beneficial effect on the sedimentation dynamics.

In concentric syringes, the outlet is located at the centre of the syringe cross-section resulting in the presence of a non-effective region in the syringe. According to the model, this effect can be eliminated by using an eccentric syringe, i.e. a syringe with the outlet in line with the edge of the syringe barrel. To quantify the resulting effect, the sedimentation dynamics of particles (${\rho }_{P}$=1.07 g/cm^3^, ${r}_{P}$= 5 μm) were simulated in syringes $({R}_{S}$ =3.6 mm) with different outlet position, namely concentric, eccentric with the outlet lined with the bottom edge of the syringe, and eccentric with the outlet lined with the top edge of the syringe. As shown in Fig. 6b, simulated concentration half-life values were ${t}_{1/2}$= 440, 580, and 410 s, respectively, resulting in a > 30% increase in ${t}_{1/2}$ for the best eccentric syringe over the standard concentric syringe.

Finally, to demonstrate how the overall choice of the syringe can significantly affect the concentration half-life ${t}_{1/2}$ of the sample, the simulated sedimentation of particles (${r}_{P}$= 5 μm, ${\rho }_{P}$=1.07 g/cm^3^) was computed for a concentric syringe with ${R}_{S}$=1.15 mm (corresponding to a 250 µl syringe) commonly used in lab-on-chip applications (e.g. Aghel 2024) and for an eccentric syringe with ${R}_{S}$=3.6 mm (corresponding to a 2.5 ml syringe). The total volume was kept constant in both simulations. As shown in Fig. 6c, the concentration half-life increased from ${t}_{1/2}$=180 s of the first configuration to ${t}_{1/2}=$ 576 s, resulting in a more than threefold increase.

### Practical guidelines for sedimentation minimization

Here, we summarize the findings obtained with the proposed model into guidelines aimed at minimizing sedimentation. These practical guidelines require minimal setup adjustments and can be used by scientist during the experiment design stage to maximize the expected lifetime of their samples:

*Buffer density*: As previously demonstrated elsewhere (e.g. Martin et al. 2006), minimizing the density mismatch between particles and buffer increases the sample lifetime by reducing driving force of the sedimentation (${t}_{1/2}\propto {\left({\rho }_{B}-{\rho }_{P}\right)}^{-1}$). Experimentally, this can be achieved by adding a solute to the buffer to achieve ${\rho }_{B}$=${\rho }_{P}$ (large molecules are recommended if biological particles are used).

*Buffer viscosity*: As previously demonstrated elsewhere (e.g. Freyer et al. 1989; Otto et al. 2015), increasing the buffer viscosity increases the sample lifetime by creating additional drag between particles and the buffer. From a modelling perspective, the higher the viscosity and the lower the sedimentation (${t}_{1/2}\propto \eta$). Nevertheless, a higher viscosity will lead to higher shear stresses potentially damaging for biological particles. Experimentally, the target buffer viscosity should be set based on the maximum tolerable shear stresses and obtained by addition of solutes such as Xanthan gum or methylcellulose.

*Sample flow-rate*: Increasing the sample flow-rate increases the sample lifetime by creating regions near the outlet where the flow is able to lift particles ($${v}_{Flow}^{y}+{v}_{T}>0)$$. From a modelling perspective, higher flow-rates increase $${t}_{1/2}$$ by bringing the sedimentation constant $${K}_{S}$$ closer to its upper boundary. Similarly to viscosity, a higher flow-rate will induce higher shear stresses on particles. The flow-rate might also be bounded by additional setup constraints (e.g. measurement sampling frequency or printing speed). Experimentally, the flow-rate should be set to the highest value tolerable by the setup.

*Syringe radius*: A larger syringe radius increases the distance that particles must travel in order to reach the bottom of the syringe, thus increasing the sample lifetime. Nevertheless, the accuracy of syringe pumps flow-rates is inversely proportional to the syringe cross-section. Experimentally the largest syringe radius ensuring a reliable flow-rate should be used.

*Syringe type (concentric/eccentric)*: The position of the syringe outlet affects the formation of non-effective regions inside the syringe. Experimentally, eccentric syringes are to be preferred over concentric syringes and used with the tip aligned with the bottom edge.

## Conclusions

In this study, we characterized the sedimentation dynamics of particles suspended inside a horizontal syringe. We developed a simple model to quantify the temporal evolution of the effective concentration, and to estimate the concentration half-life ${t}_{1/2}$, i.e. the time required for the concentration to halve. In the model, sedimentation depends on 3 main parameters, namely particle terminal velocity ${v}_{T}$, syringe radius ${R}_{S}$, and a sedimentation constant ${K}_{S}$, which is bounded in the range [0.4, 0.8] and increases with increasing flow-rate. The model was successfully tested with FEM simulations and validated with experimental measurements on polymeric particles and mammalian cells. The model was used to provide guidelines for the design of experimental setups involving particles in suspensions loaded into a horizontal syringe with minimal sedimentation.

## Supplementary Information

Below is the link to the electronic supplementary material.

Supplementary file1 (DOCX 1603 KB)

## Data Availability

Data generated and analyzed in this study are available at the online repository: https://github.com/RealeRiccardo/Sedimentation_Horizontal_Syringe.
