# Supplementary material for: Modelling, simulation, and experimental characterization of particle sedimentation inside a horizontal syringe
Source: Microfluid Nanofluidics. 2025 Apr 15;29(5):28. doi: 10.1007/s10404-025-02802-x (PMC12000163; doi:10.1007/s10404-025-02802-x)
Supplement: Supplementary file 1 — Supplementary file1 (DOCX 1603 KB) [file 10404_2025_2802_MOESM1_ESM.docx]

**Supplementary material**


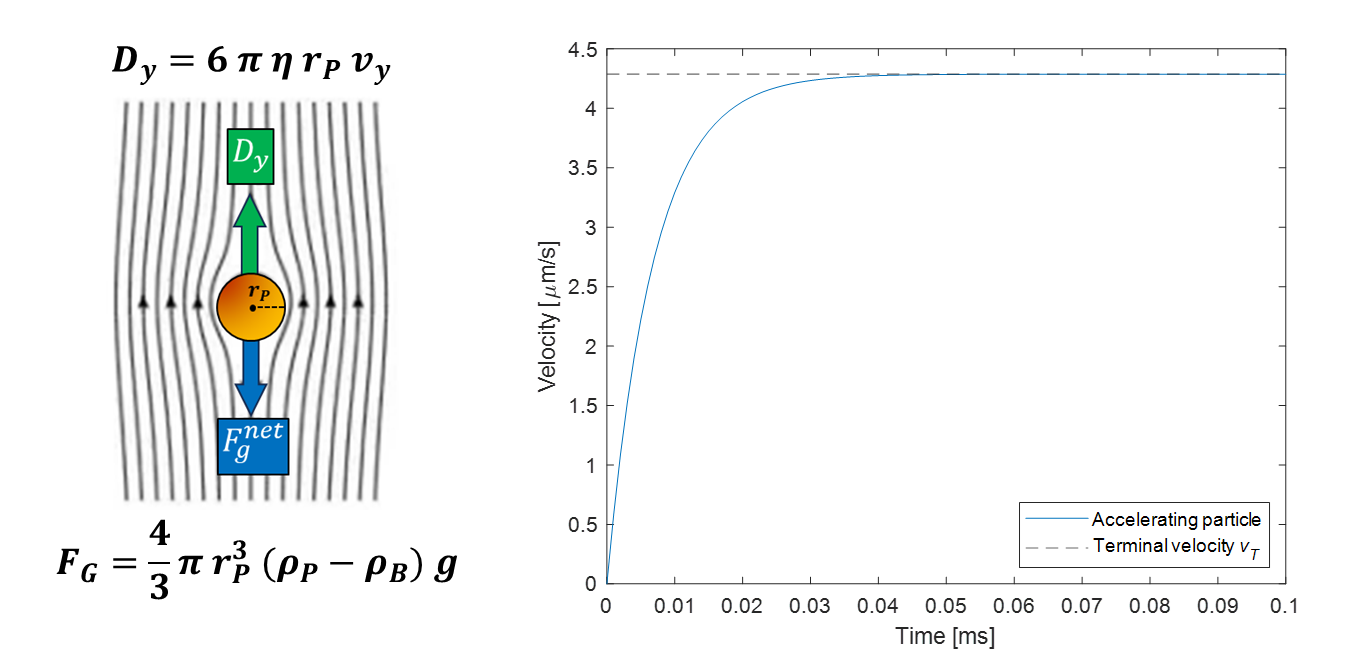


**Figure S1 –** Typical acceleration curve of a particle undergoing sedimentation. The particle is subject to gravitational acceleration and Stoke’s drag. The velocity over time was computed numerically for a particle with $\rho_{P}$=1060 kg/m^3^, $r_{P}$=5 μm and a buffer with $\rho_{B}$=997 kg/m^3^ and $\eta$=0.8 g/(m s).


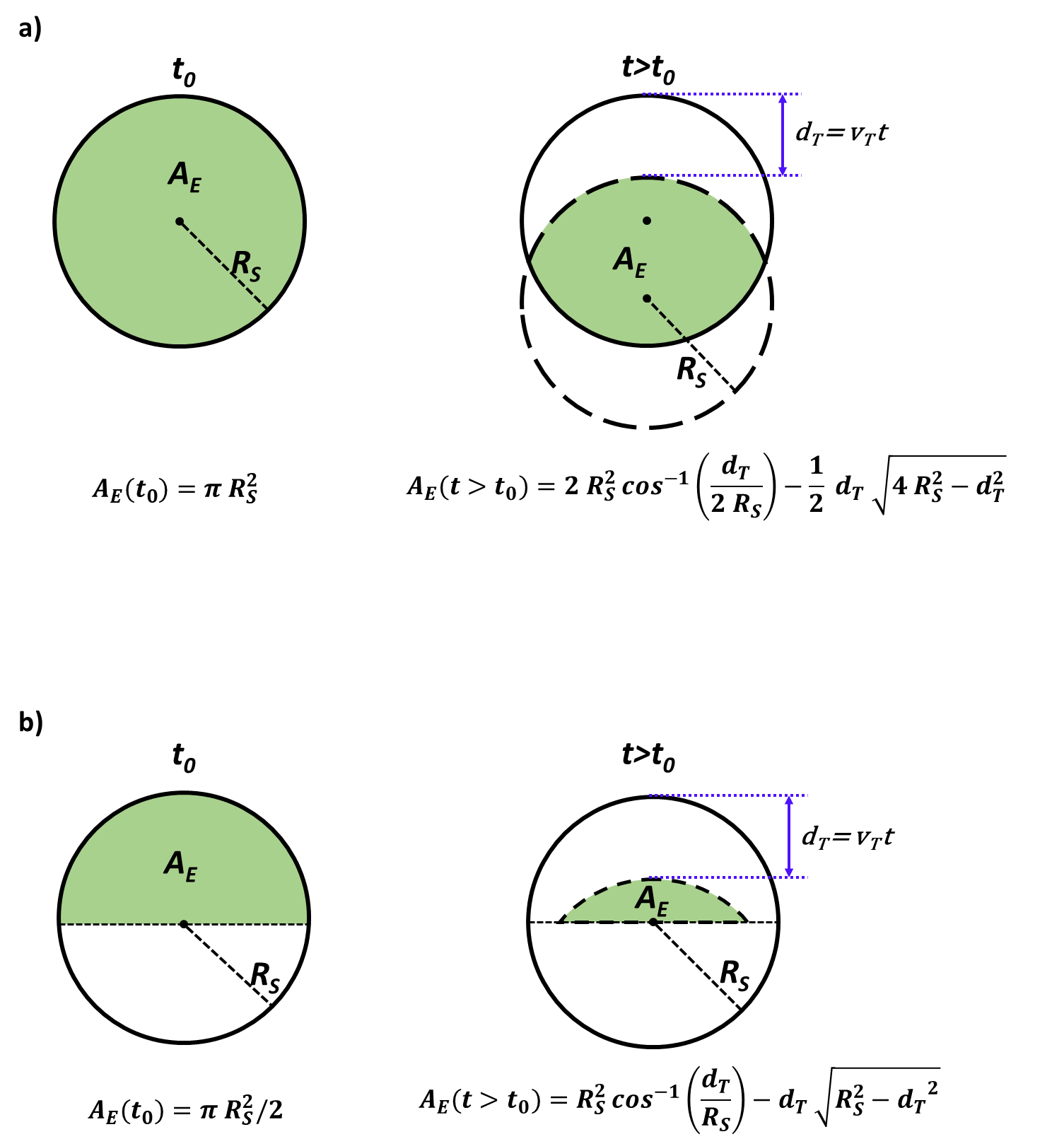


**Figure S2 –** Evolution of effective area during sedimentation. For t>$t_{0}$, particles will have travelled a distance $d_{T}$=$v_{T} t$. a) Intersection area of two circles with the same radius, $R_{S}$, with a distance $d_{T}$ between their centers. b) The area of a circular segment with chord height $R_{S}-d_{T}$ can be obtained using trigonometry.


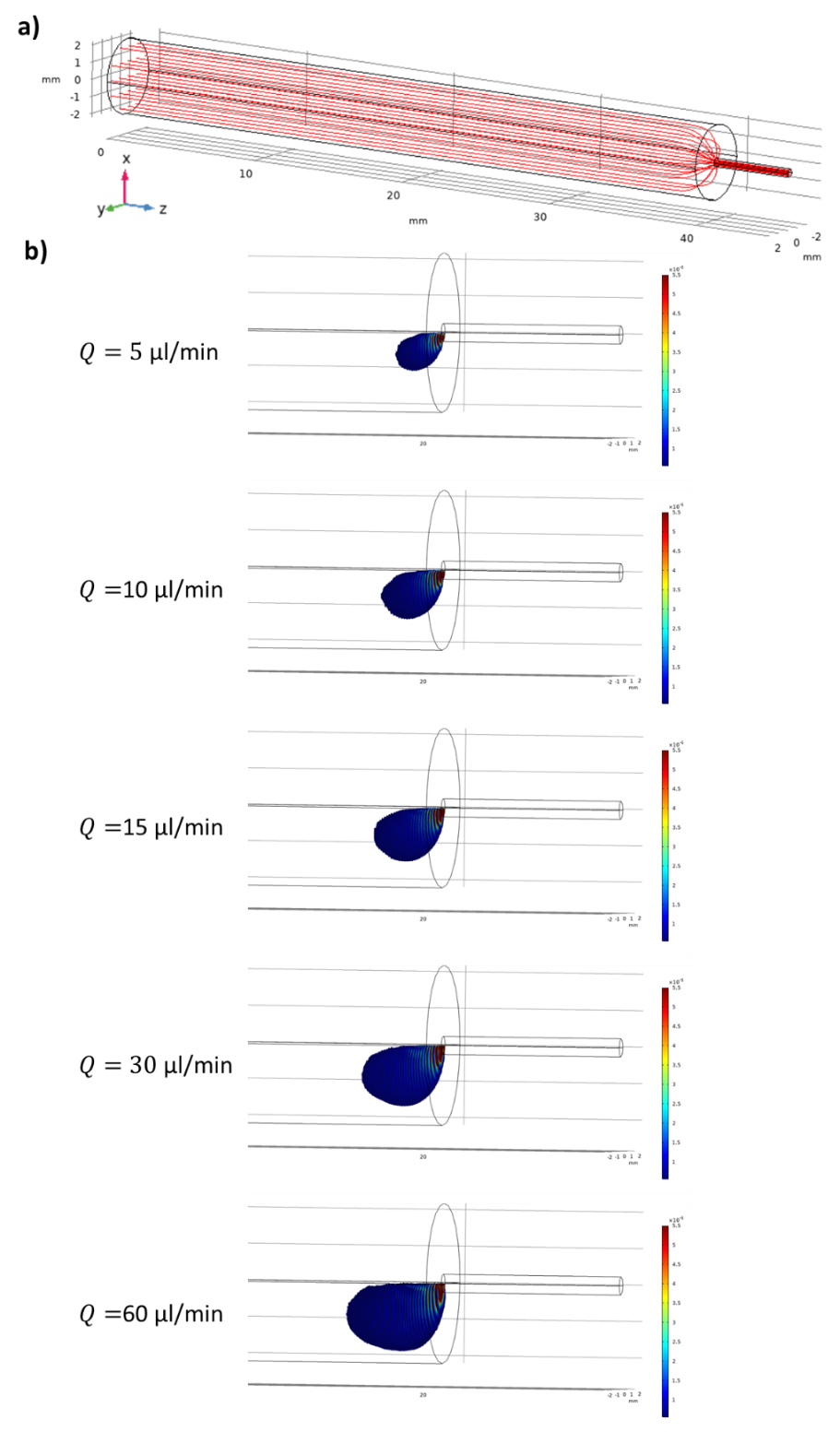


**Figure S3 –** Flow profile inside a syringe computed using FEM simulations. a) Streamlines are parallel over a large section of the syringe and converge towards the centre near the outlet. In this region, the fluid flow can lift particles if its vertical velocity is greater than particle terminal velocities ($v_{T}+ v_{Flow}^{y}>0$), thus affecting the sedimentation process. b) The presence and extent of these regions in the syringe where are difficult to compute without tailored FEM simulations. Here, regions where the condition holds true are highlighted for increasing flow-rates Q, with the color representing the vertical velocity component of the fluid flow.


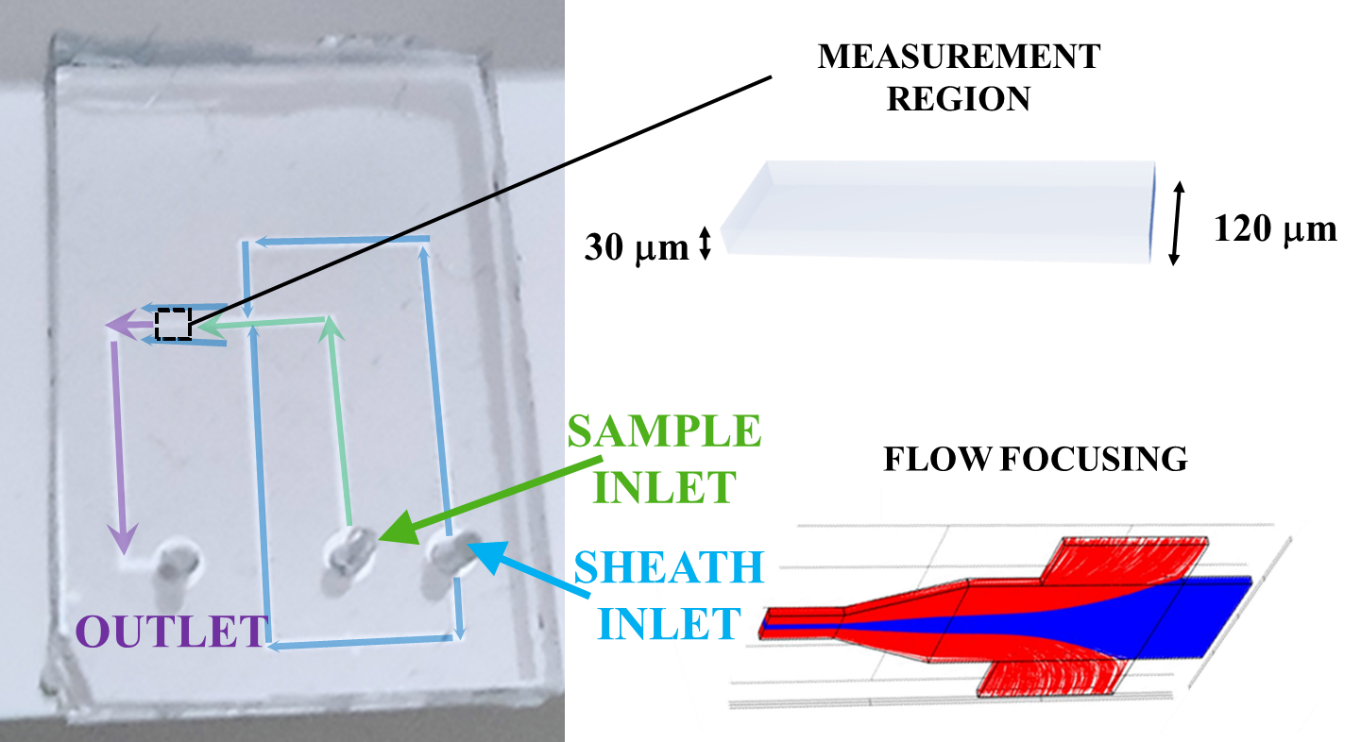


***Figure S4 –*** *Microfluidic chip used for the particle counter. The sample is pumped through the inlet, through the measurement region, to the outlet. A sheath flow (red streamlines) confines the samples (blue streamlines) in the center of the measurement region to increase the signal-to-noise ratio of the measurement. The chip is made of PDMS using standard photolithography technique.*


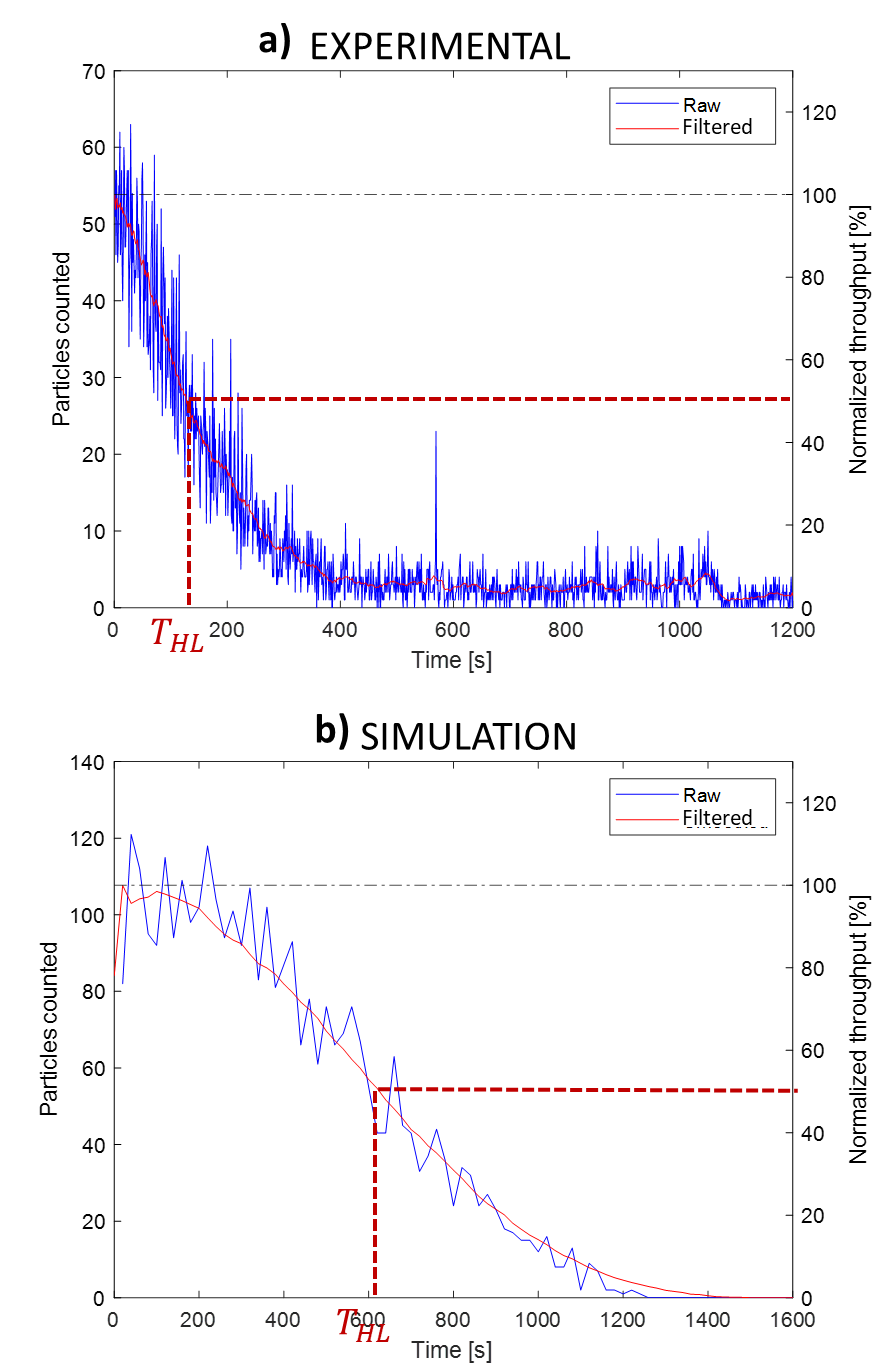


**Figure S5** – Experimental and simulation throughput curve are normalized with respect to the initial value, filtered with a low-pass filter, and the concentration half-life $t_{1/2}$ measured.


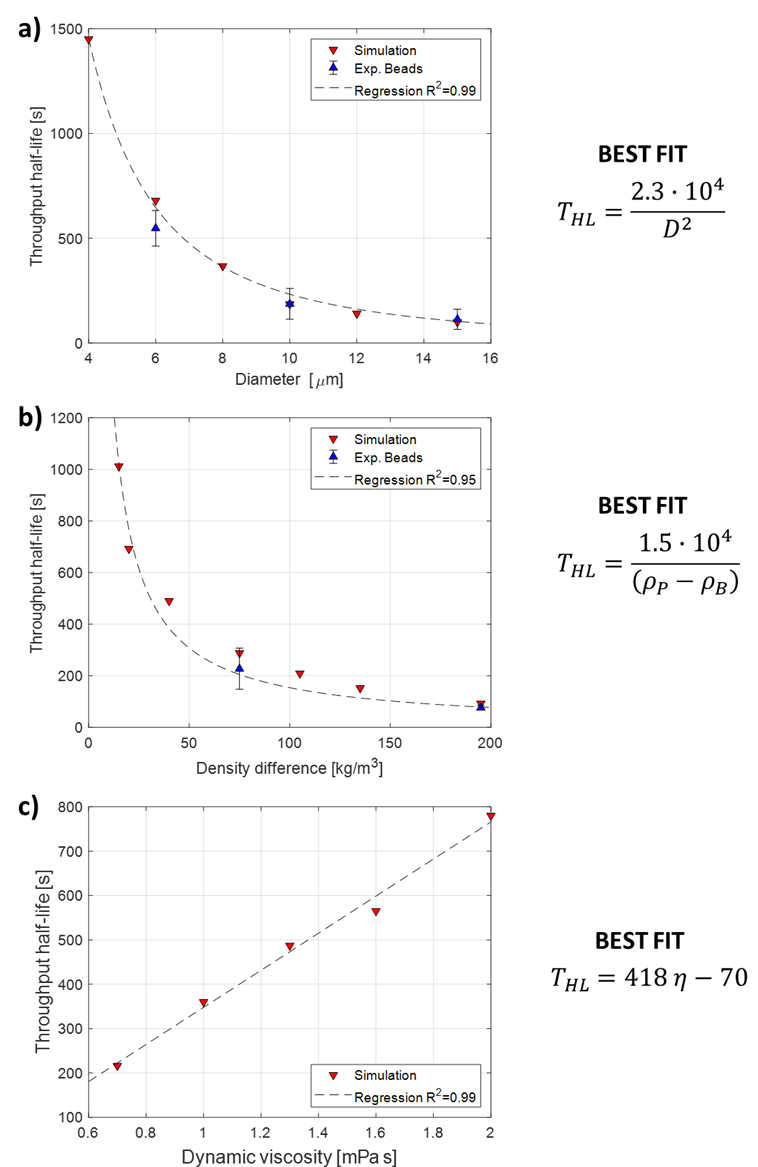


**Figure S6** – Regression curves of concentration half-life $t_{1/2}$ as a function of a) Particle diameter, b) Particle-buffer density difference, and c) Buffer dynamic viscosity.
